# ISNS Celebrates International Neonatal Screening Day 2024 with a New *IJNS* CiteScore, a Global Report and a WHO Resolution

**DOI:** 10.3390/ijns10020039

**Published:** 2024-06-13

**Authors:** Peter C. J. I. Schielen

**Affiliations:** Office-International Society for Neonatal Screening, Reigerskamp 273, 3607 HP Maarssen, The Netherlands; peter.schielen@isns-neoscreening.org

The *International Journal of Neonatal Screening* (*IJNS*), founded in 2015 by the International Society for Neonatal Screening (ISNS), has quickly become the most important journal for scientific papers on neonatal screening, as indicated by an impressive impact factor of 3.5 granted in 2023, in its ninth year. As of June 2024, our CiteScore is 6.7.

On 25 May 2024, *IJNS* encountered a new historical moment. The paper, ‘Current Status of Newborn Bloodspot Screening Worldwide 2024: A Comprehensive Review of Recent Activities (2020–2023)’ was published, with 184 pages of extensive information on the topic:

*“Therrell, B.L.; Padilla, C.D.; Borrajo, G.J.C.; Khneisser, I.; Schielen, P.C.J.I.; Knight-Madden, J.; Malherbe, H.L.; Kase, M. Current Status of Newborn Bloodspot Screening Worldwide 2024: A Comprehensive Review of Recent Activities (2020–2023). Int. J. Neonatal Screen. **2024**, 10, 38. https://doi.org/10.3390/ijns10020038.”*

The ISNS is greatly indebted to ISNS Guthrie award recipient and ISNS-founding member Bradford Therrell for composing this opus that will be valuable to neonatal screening globally for many years to come. The ISNS extends that gratitude to the co-authors and to the acknowledged ISNS members that reviewed parts of this comprehensive document.

The significance of this contribution to *IJNS* should not be underestimated. It illustrates the similarities and differences of neonatal screening programs across the world. It is the core of the mission and vision of the ISNS to evaluate that global state of neonatal screening, to appreciate those differences and identify areas for improvement to secure equitable neonatal screening and equal health outcomes for children and their parents globally.

If a summary covers on average 10% of a paper, a summary of this paper would be 18 pages long, which is possibly not palatable for our readers. So, below are a short list of teasers that will hopefully invite you to go through the pages and discover the personal neonatal screening findings of your interest.

- This paper contains an impressive 1590 citations (scientific publications, websites, reports, policy drafts); 400 from North America, 260 from the Asia-Pacific region, 435 from Europe, 194 from Latin America and the Caribbean, 146 from the Middle East and Northern Africa and 148 from Sub-Saharan Africa.
- Summarized in a table are demographic data and data on screening programs per country and a geographical map of the various regions—a valuable combination. You are invited to discover the opportunities and challenges in these data; screening programs appear where you do not expect them, are run by colleagues that might need your help and expertise, or colleagues that are eager to learn or share their experience. These data invite you to reach out, learn, wonder and share.
- With that, we realize that steps still need to be taken concerning the penetration and resolution of the datasets. We have data from every province of Canada, and we should strive to also present data from individual provinces of China and India. It is too easy to generalize, while there is so much diversity in regions, countries and provinces.
- This report is forward-looking, with pages 11–14 dedicated to what the future might have in store for the Unites States.
- For the Asia Pacific region, this report has quite extensive data from India, China, that are amidst a period of expansion of screening panels and growth, and Japan and the Philippines, that have programs with a longer history. It is interesting to see what will happen in, for instance, Indonesia, that has a strong ambition to develop robust screening programs, and in Thailand and Vietnam.

Next, the ISNS will use the wealth of data in ‘Current Status of Newborn screening worldwide’ to populate the virtual maps on the ISNS website, available for free for all ISNS members (https://membership.isns-neoscreening.org/charts/globe).

Coincidentally, Dr. Anshu Banerjee, the Director for Maternal, Newborn and Child Health at the World Health Organization, managed to have a paragraph on neonatal screening approved as part of the resolution for global policy implementation during the 77th World Health Assembly in Geneva, Switzerland (27 May–1 June 2024). 

The paragraph is as follows:

*() INVITES Member States, in accordance with national context and priorities:*

*() to consider implementing a universal newborn screening programme including comprehensive birth defect screening, including specific needs and considerations for diagnosis, management and long-term care of children with birth defects.*

(https://apps.who.int/gb/ebwha/pdf_files/WHA77/A77_ACONF5-en.pdf, p. 5).

This means that countries, non-governmental organizations, development agencies and development banks will now consider prioritizing neonatal screening among other Mother and Child Health projects.

This recognition of universal newborn screening as an important public health service at the highest WHO level is of vital importance for the work of the ISNS and all involved in neonatal screening. To have published the sharpest picture of global neonatal screening at the same time seems only fitting and is an inspiration to jointly pursue and develop the best screening practices for all babies and parents globally.

We will celebrate all this and more on 28 June 2024, the fourth International Neonatal Screening Day (https://neonatalscreeningday.org/).

